# Factors associated with hypertension among patients with type 2 diabetes: evidence from a resource-limited setting—Southern Ethiopia

**DOI:** 10.3389/fendo.2025.1534852

**Published:** 2025-08-01

**Authors:** Begidu Yakob, Eskinder Israel, Mesfin Menza Jaldo, Awoke Abraham, Tagese Yakob

**Affiliations:** ^1^ Montioring and Evaluation, Wolaita Zone Health Department, Wolaita Sodo, Ethiopia; ^2^ School of Public Health, College of Health Science and Medicine, Wolaita Sodo University, Wolaita Sodo, Ethiopia; ^3^ School of Public Health, College of Medicine and Health Sciences, Wachemo University, Hossana, Ethiopia; ^4^ School of Public Health, Walailak University, Nakhon Si Thammarat, Thailand

**Keywords:** hypertension, type 2 diabetes mellitus, primary healthcare, Ethiopia, resource limited setting

## Abstract

**Background:**

Globally, non-communicable diseases contribute to about three-quarters of all deaths. Developing nations face particular difficulties with the concurrent presence of diabetes and high blood pressure, problems intensified by poor nutrition habits, lack of physical exercise, and continuing high rates of illnesses like HIV/AIDS and malaria. A significant data gap in primary healthcare (PHC) data has been determined and prioritized by governments seeking to assess and enhance the prevention and management of chronic disease.

**Objective:**

The main aim of the current study is to determine factors related to hypertension among patients with type 2 diabetes in public PHC facilities in Southern Ethiopia.

**Methods:**

A facility-based cross-sectional study was conducted from 6 March 2023 to 5 April 2023 among 409 patients with diabetes. A systematic random sampling technique was employed to select the study participants. The data were cleaned, coded, and entered into Epi-data version 4.6.0.2 and exported to STATA version 14 for analysis. For descriptive statistics, both bivariate and multivariable logistic regression analyses were employed. Variables with a *p*-value <0.05 in the multivariable logistic regression analysis were declared as significantly associated with outcome variables.

**Results:**

A total of 407 patients with type 2 diabetes mellitus (T2DM) were included in the current study with a response rate of 99.5%, and the mean age of the study participants was 57.1 years (SD ±9.91). The prevalence of hypertension among patients with T2DM was 66.1% with a 95% confidence interval (CI) of 59.9–70.3. The age group of >60 years [adjusted odds ratio (AOR) = 2.09, 95% CI (1.02–4.28)], patients with ≥10 years’ duration of T2DM [AOR = 1.79, 95% CI (1.05–3.03)], body mass index ≥25 kg/m^2^ [AOR = 4.19, 95% CI (2.10–8.33)], and patients who have a family history of hypertension [AOR = 11.73, 95% CI (5.82–23.66)] were significantly associated with hypertension among patients with type 2 diabetes.

**Conclusion:**

The prevalence of hypertension is high and the majority has poor blood pressure control. Hence, DM care providers and other health sector stakeholders have to work in collaboration to prevent it by designing appropriate strategies especially for those at higher risk of developing hypertension.

## Introduction

High blood pressure stands as the third-leading cause of health burden measured in disability-adjusted life years (DALYs). While impacting 972 million adults globally in 2000, projections indicate this may rise to 1.56 billion cases by 2025 (1). Low- and middle-income countries (LMICs) bear 60% of the total impact, reflecting system healthcare and preventive care challenges (1). In sub-Saharan Africa (SSA), the burden reaches up to 38% in some communities, with an estimated 10–20 million hypertensive individuals among 650 million people in SSA (2–4). In Addis Ababa, Ethiopia, hypertension (HTN) burden ranges from 155 to 305, indicating significant regional variability and underdiagnosis (5).

Diabetes mellitus (DM), a chronic condition marked by hyperglycemia, affected 460 million individuals globally in 2019 and categorized as the world’s eighth-leading disability-adjusted fatal disease and ranking as the eighth-leading cause of disability-related mortality (6). By 2021, the International Diabetes Federation (IDF) reported 537 million cases, with health expenditures nearing $966 billion. Undiagnosed cases are rampant, particularly in LMICs (7, 8). In 2022, approximately 54 million adults aged above 18 years in the WHO Africa region had diabetes, with >50% undiagnosed (34 million untreated), exacerbating complications like renal failure and amputation (9). National prevalence was 3.2% in Ethiopia in 2015 by the WHO survey (10).

DM and HTN are worldwide public health issues (11). These have been shown to be two of the main risk factors for cardiocerebrovascular illnesses, which are the top causes of adult mortality and disability (12). DM and HTN are additional problems facing the healthcare system in LMICs (13), attributable to shifts in dietary patterns and physical inactivity (14, 15), and with malaria and HIV on the rise, this region faces a growing dual burden, resulting in significant public health consequences (16). This disease is disproportionately more common in countries with limited resources and underdeveloped healthcare systems (17). In underdeveloped nations, the death rate is increased by 7.2 times when diabetes and HTN coexist. According to epidemiological research, DM increases the risk of HTN (18, 19). An American research, for example, found that up to three-fourths of adults with diabetes also had HTN (20), and in China, approximately 15 million people have both conditions (21).

Type 2 diabetes mellitus (T2DM) and HTN together are especially deadly and greatly increase a person’s cardiovascular risk. High BP and T2DM also raise the risk of acquiring additional diabetes-related conditions, such as renal disease and retinopathy, which can result in blindness (22). Furthermore, the formation of resistant HTN is linked to their cohabitation (23, 24). To reduce the risk of diabetic complications, early identification of HTN and related cardiovascular risk factors is necessary (25). The treatment of HTN’s side effects, such as dialysis, cardiac bypass surgery, and stroke prevention, is an expensive intervention that depletes both private and public funds (26). Early identification and effective management of HTN are associated with significant health and financial benefits (27).

Approximately 4 out of 10 (41%) patients had at least one of the following seven chronic conditions: diabetes, asthma, HTN, and others (28). Effectively managing patients with many chronic diseases in primary care is crucial, as the prevalence of multimorbidity rises with age (29). A significant data gap in primary healthcare (PHC) data has been determined and prioritized by governments seeking to assess and enhance the prevention and care of chronic diseases (5, 30). To effectively prevent and control HTN, PHC settings need to collect comparable and aggregated data on the disease burden from the condition. This study aims to determine factors associated with HTN among patients with T2DM in Southern Ethiopia.

## Materials and methods

### Research framework, duration, and location

This cross-sectional study enrolled 409 patients with diabetes attending primary hospitals in the Wolaita zone between 6 March and 5 April 2023. The Wolaita zone is located in the southern region of Ethiopia, which is 380 km away from Ethiopia’s capital, Addis Ababa. In the study area, there exist eight primary hospitals providing services to all zonal districts’ population and other population groups near the Wolaita zone.

### Population

The study included adult patients with DM aged ≥18 years who were under follow-up at selected public PHC chronic outpatient departments and met the inclusion criteria. Eligible participants had a documented history of at least three consecutive follow-up visits at the study facility. Critically ill patients and pregnant women with DM were excluded during the data collection period.

### Sample size determination and sampling procedure

The sample size (*n*) was calculated using the single population proportion formula, assuming an HTN prevalence of 59.5% among patients with T2DM of another study conducted at DMGH (31) with 95% confidence interval (CI) and 5% margin of error, and 10% of the population size was added to adjust the non-response rate of study participants and to estimate our study sample size. Thus, $sample\;size=\frac{{\left(Z\frac{a}{2}\right)}^{2}*\text{ p}\left(1-\text{p}\right)}{d^{2}}\;=\;\frac{{\left(1.96\right)}^{2}*\;0.595\left(1-0.595\right)}{0.05^{2}}n\;=\;370$ , and after adding 10% of the non-response rate, the sample used for final study was *n* = 407.

The study was conducted at seven randomly selected primary hospitals (Tebela, Bele awassa, Bodit, Bedesa, Gesuba, Bitana, and Bomb primary hospital) across the study setting. An SRS method was used to identify the study subjects and 81 adult patients with DM who were followed at Tebela primary hospital, 85 at Bele awassa primary hospital, 92 at Bodit primary hospital, 69 at Gesuba primary hospital, 48 at Bitana primary hospital, 41 at Bedesa primary hospital, and 65 at Bomb primary hospital. The patient’s medical registration number from each primary hospital HTN registration log book was first used to establish a sampling frame. Following that, each hospital received a proportionate share of the computed sample size. Next, a computer-generated specialized random sampling procedure was used to choose research participants from each of the chosen hospitals.

### Operational definition

HTN: Participants were classified as hypertensive if they met either of the following criteria: (1) average systolic blood pressure (SBP) ≥140 mmHg and/or diastolic blood pressure (DBP) ≥90 mmHg, or (2) current use of antihypertensive medication (32). HTN staging followed standard classifications: prehypertension (120–139/80–89 mmHg), stage 1 (140–159/90–99 mmHg), stage 2 (160–179/100–109 mmHg), and hypertensive crisis (≥180/110 mmHg) (33). Current smokers were defined as individuals who reported cigarette use at least once during the month preceding the study (34). Poor glycemic control was operationally defined as having a hemoglobin A1c (HbA1c) level exceeding 7% (35).

Patients with diabetes were identified through medical records as individuals with an established diagnosis who were actively engaged in follow-up care at the participating healthcare facilities, as evidenced by possession of a medical record number.

### Data collection instrument and procedure

Data were collected using a pretested interviewer-administered questionnaire adapted from relevant literature (31, 36). The study employed a mixed-method approach involving face-to-face interviews, physical examinations, and medical record reviews. Behavioral risk factors were evaluated using the WHO STEPwise surveillance methodology for chronic disease risk factors. Anthropometric measurements included weight, measured to the nearest 0.1 kg using a calibrated scale, and height, measured in meters using a stadiometer with participants standing erect on a level surface. Body mass index (BMI) was subsequently calculated as weight in kilograms divided by height in squared meters.

Blood pressure measurements were obtained using a manual mercury sphygmomanometer, with the left arm positioned at heart level. Participants rested for at least 10 min before measurement (extended to 30 min for those who consumed hot beverages like coffee). Three consecutive readings were taken, and the average of these measurements was used for analysis.

To ensure data quality, the questionnaire was pretested on 5% of the total sample size at a non-selected primary hospital, with reliability assessed using Cronbach’s alpha. Data collection was conducted by four nurses holding bachelor’s degrees, who received 1 day of training on fundamental data collection principles. Supervisors underwent additional training focused on ensuring data completeness, cross-checking entries, and error correction.

### Data analysis

The collected data were cleaned using Epi-Data software version 4.6.0.2 before being exported to SPSS version 26 for statistical analysis. Initial analysis involved computation of descriptive statistics, with results presented as frequencies and percentages. Associations between explanatory and outcome variables were examined using bivariate and multivariable logistic regression with a stepwise approach. Statistical significance was determined by an adjusted odds ratio (AOR) with a 95% CI and a *p*-value of less than 0.05.

Data normality was assessed through visual inspection of Q-Q plots and histograms, with the Q-Q plot approximating the diagonal line and the histogram showing no skewness, confirming normal distribution. Multicollinearity among predictor variables was evaluated using tolerance values greater than 0.1 and variance inflation factors (VIFs) less than 10, indicating no significant collinearity. Model fitness was assessed using the Hosmer–Lemeshow goodness-of-fit test, which yielded a *p*-value of 0.501 (χ² = 1.44), confirming that the model was well-fitted to the data.

### Ethics statement

The Ethical Review Committee (ERC) of the College of Health Science, School of Public Health, Wachemo University performed a review and allowed the study to proceed (ref. no. wcu/000981/15). In addition, formal letters of permission were obtained from the Tebela town administrative health office, the Bele awassa town administrative health office, the Bodit town administrative health office, the Bedesa district health office, the Gesuba town administrative health office, the Bitana district health office, and the Bomb district health office. The letters of permission written from district/town health offices were submitted to the respective hospitals. The study’s objectives and methods were explained to each participant. Additionally, prior to participation, all study participants provided written informed permission. The study’s goal, the thorough review of their medical records, and the potential benefits of the research were also explained to the participants. The data gathered were used for the study’s objectives. All of the information gathered throughout the study was kept private, available only to the research team and utilized only for that reason. The participants’ medical record numbers were utilized instead of personal identifiers for the purpose of gathering data. The study was carried out in compliance with the applicable rules, laws, and Helsinki Declaration principles.

### Patient and public involvement

No patients or public groups were involved in the design, analysis, or interpretation of this study, and they were also not involved in the dissemination of the results.

## Results

### Socio-demographic characteristics of study participants

The study included 407 out of 409 eligible patients with T2DM, yielding a response rate of 99.5%. The mean age of the participants was 57.1 years (± 9.91 SD), with the largest age group (35.6%, *n* = 145) being 51–60 years old. The cohort consisted of predominantly male (52.1%, *n* = 212) and urban-dwelling (85.7%, *n* = 349) individuals. Educationally, 41.0% (*n* = 167) had attained education beyond a college diploma, while 73.0% (*n* = 297) were married. Government employees constituted 39.6% (*n* = 161) of participants, and over half (52.8%, *n* = 215) reported a monthly income exceeding 5,000 Ethiopian birr (ETB). These characteristics collectively describe an urban-predominant, middle-aged population with relatively high educational attainment and income levels compared to national averages (**Table 1**).

**Table 1:** Socio-demographic characteristics of patients with type 2 diabetes mellitus: evidence from a resource-limited setting—Southern Ethiopia.

Variables	Response category	Hypertension		Total (N)
		Yes (%)	No (%)	
Age of mother (in years)	<50	70 (26.0)	49 (35.5)	119 (29.2)
	51–60	87 (32.3)	58 (42.0)	145 (35.6)
	>60	112 (41.6)	31 (22.6)	143 (35.1)
Sex	Male	140 (52.1)	72 (52.2)	212 (52.1)
	Female	129 (47.9)	66 (47.8)	195 (47.9)
Marital status	Single	18 (6.9)	12 (8.6)	10 (1.1)
	Married	186 (69.1)	111 (80.4)	364 (95.4)
	Others*	65 (24.2)	15 (10.8)	32(3.5)
Educational status	Illiterate	23 (8.6)	13 (8.8)	36 (8.8)
	Literate	12 (4.5)	19 (12.8)	31 (7.6)
	Primary	45 (16.7)	21 (14.2)	66 (16.2)
	Secondary	64 (23.8)	43 (29.1)	107 (26.3)
	College/university diploma or above	125 (46.5)	52 (35.1)	167 (41.0)
Occupation status	Merchant	27 (10.0)	13 (8.5)	35 (8.6)
	Government employee	98 (36.4)	73 (47.8)	161 (39.6)
	Housewife	73 (27.1)	29 (18.9)	102 (25.1)
	Farmer	29 (10.8)	15 (9.8)	44 (10.8)
	Others**	42 (15.6)	23 (15.0)	65 (16.0)
Residents	Urban	232 (86.3)	117 (59.1)	349 (85.7)
	Rural	37 (13.7)	21 (40.9)	58 (14.3)
Monthly income (ETB)	<1,599	14 (5.2)	42 (30.2)	55 (13.5)
	1,600–3,599	19 (7.1)	9 (6.5)	28 (6.9)
	3,600–4,999	65 (24.2)	44 (31.7)	109 (26.8)
	≥5,000	171 (63.5)	44 (31.7)	215 (52.8)

*Divorced and widowed. **Driver, security, and self-employee. ETB, Ethiopian birr.

### Clinical characteristics of participants

The study population had an average diabetes duration of 10.8 years (± 3.68 SD), ranging from 3 to 25 years. Glycemic control measurements showed a mean HbA1c of 8.2% (± 2.37 SD), with 54.1% (*n* = 220) of participants exhibiting poor glycemic control (HbA1c >7%). The average fasting blood glucose level was 152.2 mg/dL (± 45.8 SD), while mean blood pressure readings were 131.4/82.5 mmHg (± 16.7/9.0 SD) for systolic and diastolic pressures, respectively. Treatment patterns revealed that 39.8% (*n* = 162) of patients were on oral hypoglycemic monotherapy, while 37.6% (*n* = 153) received dual therapy for diabetes management. Anthropometric data indicated that 66.1% (*n* = 269) of participants fell within the normal BMI range (18.5–24.9 kg/m²). These metabolic parameters collectively demonstrate the clinical characteristics and management profile of the studied diabetic population. Approximately 281 (69.05%) study participants had no history of HTN in the family. Majority of study participants, 403 (99.0%), had no history of chronic kidney disease (CKD), 375 (92.1%) had no history of cardiac heart disease (CHD), 376 (92.4%) had no history of alcohol drinking, and 395 (97.1%) had no history of smoking. Greater than half, 219 (53.8%), of study participants had physical activity of less 3 days per week. Greater than two-thirds, 274 (67.3%), of participants had a history of diabetes for more than 10 years’ duration and 293 (72.0%) participants had a self-glucometer at home. Majority of respondents, 405 (99.5%), had a history of attending health education on DM at a health facility (**Table 2**).

**Table 2:** Clinical characteristics of patients with type 2 diabetes mellitus: evidence from a resource-limited setting—Southern Ethiopia.

Variables	Response category	Hypertension		Total (*N*)
		Yes	No	
Hypertensive history with anti-hypertensive therapy	Yes	146 (54.3)	55 (39.9)	201 (49.4)
	No	123 (45.7)	83 (60.1)	206 (50.6)
If yes, how long (*n* = 201)	<10 years	108 (77.1)	55 (87.3)	163 (40.0)
	≥10 years	32 (22.9)	6 (12.7)	38 (9.3)
Current SBP in mmHg	90–119	32 (11.9)	33 (23.9)	65 (16.0)
	120–139	110 (40.8)	67 (48.6)	177 (43.5)
	140–159	112 (41.6)	26 (18.8)	138 (33.9)
	160–179	13 (4.8)	10 (7.2)	23 (5.7)
	≥180	2 (0.7)	2 (1.4)	4 (1.0)
Current DBP in mmHg	60–79	37 (13.8)	60 (44.5)	97 (23.8)
	80–89	108 (40.1)	33 (24.5)	141 (34.6)
	90–99	123 (45.7)	43 (31.9)	166 (40.8)
	≥100	1 (0.4)	2 (1.5)	3 (0.7)
Hypertensive history in the family	Yes	114 (42.4)	12 (8.7)	126 (31.0)
	No	155 (57.6)	126 (91.3)	281 (69.0)
History of CKD	Yes	3 (0.01)	1 (0.7)	4 (1.0)
	No	266 (99.9)	137 (99.3)	403 (99.0)
History of CHD	Yes	30 (7.9)	2 (1.4)	32 (7.9)
	No	239 (92.1)	136 (98.6)	375 (92.1)
Smoking history	Yes	7 (0.8)	5 (3.6)	12 (2.9)
	No	262 (97.2)	133 (96.4)	395 (97.1)
Alcohol intake	Yes	15 (5.6)	16 (11.5)	31 (7.6)
	No	254 (94.4)	122 (88.5)	376 (92.4)
Physical activity status/week	<3 days	114 (42.4)	60 (43.5)	219 (53.8)
	3–5 days	155 (57.6)	58 (42.0)	138 (33.9)
	>5 days	114 (42.4)	20 (14.5)	50 (12.3)
BMI (kg/m^2^)	<25	147 (54.6)	122 (88.4)	269 (66.1)
	≥25	122 (45.3)	16 (11.6)	138 (33.9)
Anti-diabetic drug use	OHA	99 (36.8)	63 (45.6)	162 (39.8)
	Insulin	59 (21.9)	33 (23.9)	92 (22.6)
	OHA + insulin	111 (41.2)	42 (30.4)	153 (37.6)
Glycemic control	Good	105 (39.0)	82 (59.4)	187 (45.9)
	Poor	164 (60.9)	56 (40.6)	220 (54.1)
Fasting glucose level	<126 mg/dL	112 (41.6)	37 (26.8)	149 (36.6)
	≥126 mg/dL	157 (58.4)	101 (73.2)	258 (64.4)
Duration of T2DM	<10 years	70 (26.1)	63 (45.6)	133 (32.7)
	≥10 years	199 (73.9)	75 (54.4)	274 (67.3)
Have glucometer at home	Yes	76 (28.3)	38 (27.5)	114 (28.0)
	No	193 (71.7)	100 (72.5)	293 (72.0)
Attending health education on DM	Yes	267 (99.2)	138 (100.0)	405 (99.5)
	No	2 (0.8)	0 (0.0)	2 (0.5)

*CKD: chronic kidney disease, CHD: cardiac heart disease, DM: diabetic mellitus, BMI: body mass index.

### Prevalence and patterns of hypertension among patients with T2DM

HTN was identified in 66.1% (95% CI: 59.9–70.3) of the participants with type 2 diabetes. From these, 68 (25.2%) were newly diagnosed at the time of data collection. The majority, 177 (43.5%), of patients with type 2 diabetes were prehypertensive, followed by stage 1 hypertensive, 138 (33.9%); 23 (5.7%) study participants were stage 2 hypertensive and 4 (1%) were in the hypertensive crisis category.

### Statistical analysis of hypertension risk factors

The association between potential predictors and HTN was examined through binary logistic regression analysis. Initial candidate variables considered for the model included age, marital status, family history of HTN, physical activity level, duration of T2DM, BMI, use of anti-diabetic medications, glycemic control status, and fasting blood glucose levels. In the final multivariate analysis, four factors emerged as statistically significant predictors of HTN: age, duration of T2DM, BMI, and family history of HTN.

Advanced age showed a significant association with HTN risk. After adjusting for all other variables in the model, participants aged over 60 years demonstrated 2.09 times greater odds of developing HTN compared to those younger than 50 years (AOR = 2.09; 95% CI: 1.02–4.28; *p* = 0.044). Elevated BMI also proved to be an important risk factor, with overweight or obese participants (BMI ≥ 25 kg/m²) having 4.19 times higher odds of HTN than those with normal BMI (AOR = 4.19; 95% CI: 2.10–8.33; *p* = 0.001).

The duration of diabetes showed a significant relationship with HTN risk. Patients with a T2DM duration of 10 years or more had 1.8 times greater odds of developing HTN compared to those with a shorter disease duration (AOR = 1.8; 95% CI: 1.05–3.03; *p* = 0.03). Most strikingly, family history of HTN emerged as the strongest predictor, with affected participants showing 11.7 times higher odds of developing HTN compared to those without such history (AOR = 11.73; 95% CI: 5.82–23.66; *p* = 0.001). The notably wide CI for family history may reflect limited precision due to the relatively small subgroup size (*n* = 126). This finding was further validated using Firth’s penalized regression analysis, which addresses small-sample bias, confirming the strong association (AOR = 10.41; 95% CI: 5.12–21.14) (**Table 3**).

**Table 3:** Multivariable logistic regression analysis of factors associated with hypertension among patients with type 2 diabetes mellitus: evidence from a resource-limited setting—Southern Ethiopia.

Variable	Hypertension	Odds ratio (95% CI)			Firth’s penalized
	Yes (%)	No (%)	COR with 95% CI	AOR with 95% CI	AOR (95% CI)*
Age (years)							
≤50	70 (26.0)	49 (35.5)	1.00	1.00	–
51–60	87 (32.3)	58 (42.0)	1.05 (0.64–1.72)	094 (0.50–1.77)	–
≥60	112 (41.6)	31 (22.6)	2.53 (1.47–4.34)	2.09(1.02–4.28)*	–
Marital status							
Single	18 (6.9)	12 (8.6)	1.00	1.00	–
Married	186 (69.1)	111 (80.4)	1.12 (0.52–0.41)	0.77 (0.29–1.99)	–
Others***	65 (24.2)	15 (10.8)	2.89 (1.15–7.26)	1.16 (0.35–3.79)	–
Family history of hypertension							
Yes	114 (42.4)	12 (8.7)	7.72 (4.07–14.6)	11.7 (5.8–23.6)*	10.41 (5.12–21.14)*
No	155 (57.6)	126 (91.3)	1.00	1.00	1.00
Physical activity (days per week)							
≤3	114 (42.4)	60 (43.5)	1.76 (0.93–3.35)	1.23 (0.55–2.76)	–
3–5	155 (57.6)	58 (42.0)	0.92 (0.47–1.77)	0.65 (0.29–1.44)	–
>5	114 (42.4)	20 (14.5)	1.00	1.00	–
Body mass index (BMI)							
<25 kg/m^2^	147 (54.6)	122 (88.4)	1.00	1.00	–
≥25 kg/m^2^	122 (45.3)	16 (11.6)	6.32 (3.56–11.2)	4.19 (2.11–8.33)*	–
Anti-diabetic drug							
OHA	99 (36.8)	63 (45.6)	1:00	1:00	–
Insulin	59 (21.9)	33 (23.9)	1.14 (0.66–1.93)	0.94 (0.48–1.80)	–
Both OHA and insulin	111 (41.2)	42 (30.4)	1.68 (1.04–2.70)	1.14 (0.64–2.0)	–
Glycemic control							
Good	105 (39.0)	82 (59.4)	1:00	1:00	–
Poor	164 (60.9)	56 (40.6)	2.28 (1.50–3.47)	0.69 (0.41–1.18)	–
Duration of T2DM							
<10 years	70 (26.0)	63 (45.6)	1 (reference)	1 (reference)	–
≥10 years	199 (73.9)	75 (54.4)	2.38 (1.55–3.67)	1.79 (1.06–3.03)*	–

COR, crude odds ratio; T2DM, type 2 diabetic mellitus. *Statistically significant at *p* < 0.05. ***Divorced and widowed.

## Discussion

This study showed the prevalence of HTN and associated factors among patients with T2DM in public PHC facilities in Southern Ethiopia. The prevalence of HTN in this study was 66.1% with a 95% CI of 59.9–70.3. The current findings are in line with studies in Botswana (63.1%) (32) and in Israel (60.2%) (5). However, the current findings are higher than the prevalence reported in Nigeria (54.2%) (33), Jimma (46.5%) (18), Hosanna (55%) (37), Debor Tabour, Ethiopia (59.5%) (31), Adama, Ethiopia (56.3%) (38), a national diabetes center in Greece (55.6%) (39), and the United States (49.8%) (40). The observed HTN prevalence (66.1%) was lower than that reported in Jordan (72.4%) (41). The substantial differences in HTN prevalence across studies may be attributed to several factors, including variations in diagnostic criteria and distinct population characteristics. Notably, a prospective cohort study conducted in Iraq from August 2008 to April 2011 involving 5,578 patients with T2DM reported an HTN prevalence of 89.6%, with 45.3% of cases being newly identified during the study period (42). The observed discrepancies may stem from variations in diagnostic thresholds (e.g., >130/80 mmHg versus higher cutoffs), differences in study populations’ sociodemographic profiles, and methodological factors including study design, healthcare access patterns, and lifestyle variations among participants. These elements collectively contribute to prevalence rate differences across studies while highlighting the importance of standardized diagnostic criteria and population-specific considerations in HTN research. Such variations underscore the need for careful interpretation of findings relative to each study’s unique context and methodology.

The prevalence of newly diagnosed HTN was 25.2% with a 95% CI of 21.7–29.0. The current findings were in line with studies conducted in Dire Dawa city, eastern Ethiopia (24.43%) (43) and in Gimbi Town, western Ethiopia (24.8%) (44). However, the HTN prevalence identified in our study substantially exceeds the 15.8% rate documented in Ethiopia’s national NCD STEPS survey of the general adult population (45), in Debre-Markos, northwest Ethiopia (12.7%) (31), and Durame town, southern Ethiopia (14%) (46). However, the current findings were lower than those in community-based studies in Addis Ababa, central Ethiopia (newly diagnosed HTN prevalence, 29.2%) (47) and a multicenter facility-based study in Addis Ababa, central Ethiopia (newly diagnosed HTN prevalence, 32.3%) (48). This discrepancy may occur because individuals at risk of HTN are more likely to seek screening at urban tertiary care centers than at PHC facilities. The increased access to specialized services in major urban areas could lead to higher case detection rates compared to peripheral health institutions.

The study demonstrated a significant age-dependent association with HTN, where participants aged >60 years had 2.09 times higher odds of developing HTN (AOR = 2.09; 95% CI: 1.02–4.28) compared to those <50 years. These findings align with previous epidemiological studies conducted in Israel (49), Iraq (42), and Botswana (50), confirming the well-established relationship between advancing age and increased HTN prevalence. The observed association can be attributed to age-related physiological and structural changes in the cardiovascular system. With progressive aging, the vascular system undergoes significant remodeling characterized by endothelial dysfunction, reduced arterial elasticity, and increased vascular stiffness. These changes result from complex pathophysiological alterations affecting all three layers of the arterial wall—the intima, media, and adventitia. Key mechanisms include diminished nitric oxide bioavailability, increased collagen deposition, elastin fragmentation, and medial calcification (51).

This study further demonstrated that individuals with a BMI of ≥25 kg/m² had a significantly elevated risk of developing HTN compared to those with normal BMI. These findings align with previous research conducted in various settings, including Jimma, Gondar, Jijiga, Bayisa State, Nigeria, Kenya, and urban Varanasi (18, 52–57). The pathophysiological basis for this association involves multiple interrelated mechanisms. Excess adiposity contributes to increased cardiovascular risk through endothelial dysfunction, chronic low-grade inflammation, and hemodynamic alterations that collectively promote atherosclerosis (58). Furthermore, overweight and obesity induce insulin resistance and dyslipidemia, characterized by elevated low-density lipoprotein cholesterol deposition in vascular walls. These metabolic disturbances lead to progressive arterial stiffening and luminal narrowing, ultimately resulting in HTN development (32).

The study demonstrated a significant duration-dependent relationship between T2DM and HTN, with longer diabetes duration correlating with increased HTN prevalence. This finding is consistent with prior research indicating that prolonged T2DM exacerbates metabolic and vascular dysfunction, thereby elevating HTN risk (16, 30, 31). The underlying mechanisms involve progressive metabolic and hemodynamic disturbances associated with chronic hyperglycemia. Extended exposure to insulin resistance, dyslipidemia, and sustained hyperglycemia leads to cumulative microvascular damage, endothelial dysfunction, and sympathetic nervous system overactivation. Additionally, prolonged diabetes duration enhances renin–angiotensin–aldosterone system (RAAS) activity while reducing insulin-mediated vasodilation, further contributing to elevated blood pressure (34).

The study demonstrated a robust association between familial HTN history and HTN development in patients with T2DM. Individuals with a family history of HTN exhibited a 10.4-fold increased likelihood of developing HTN compared to those without such history (AOR = 10.4, 95% CI: 5.12–21.14). These findings align with epidemiological evidence from Sri Lanka (58), Northern India (59), and multiple international studies (60–64). The observed familial aggregation likely reflects both shared genetic susceptibility and common environmental exposures. From a genetic perspective, multiple polygenic variants affecting renal sodium handling, vascular tone regulation, and RAAS system function may contribute to this predisposition (26). Additionally, familial clustering of modifiable risk factors—including dietary patterns (particularly sodium and potassium intake), physical activity levels, and stress coping mechanisms—may synergistically interact with genetic predisposition to elevate HTN risk within families.

### Limitation of the study

While this study identified four significant predictors of HTN (age, family history, BMI, and diabetes duration), several potential confounding variables remain unaccounted for in our analysis. These include medication adherence patterns, specific dietary factors (such as sodium intake or DASH diet compliance), and objective measures of physical activity. Such unmeasured variables may represent important modifiers of HTN risk that could influence the observed associations. Future research incorporating these additional parameters would provide a more comprehensive understanding of HTN risk factors in diabetic populations.

## Conclusion

This study revealed that over 50% of participants with T2DM also had HTN. Our analysis identified significant associations between HTN and four key factors: advanced age, family history of HTN, elevated BMI, and longer duration of T2DM.

Understanding the global prevalence of HTN in diabetic populations is crucial for formulating effective public health strategies. Such epidemiological data inform both national and international healthcare policies aimed at managing this dual disease burden. HTN and diabetes are well-established, independent risk factors for atherosclerotic cardiovascular diseases, including coronary artery disease and cerebrovascular accidents. When these conditions coexist, they synergistically amplify the risk of serious complications, creating a greater combined effect than either condition alone. Medical practitioners ought to recommend regular blood pressure checks for patients’ relatives and promote preventative measures including dietary improvements and increased physical activity. The Ministry of Health and other concerned policymaker bodies should enhance care through workforce training and support interdisciplinary teams of primary care providers, fund community-based screening approaches, integrate HbA1c testing into routine primary care, develop guidelines prioritizing intensive lifestyle program for patients with overweight and family history of the disease, and continue health advocacies and promote lifestyle modification. Furthermore, future studies should highlight the need for more comprehensive longitudinal studies with detailed risk factor assessment.

## Data Availability

The raw data supporting the conclusions of this article will be made available by the authors, without undue reservation.
